# Surgical Management During the COVID-19 Era at a Private Tertiary Care Hospital of Karachi, Pakistan: A Cross-Sectional Study

**DOI:** 10.7759/cureus.21012

**Published:** 2022-01-07

**Authors:** Nida Zahid, Erum Baig, Gulzar Lakhani, Hasnain Zafar, Asad Latif, Syed Ather Enam

**Affiliations:** 1 Surgery, Aga Khan University Hospital, Karachi, PAK; 2 Anesthesia and Critical Care, Aga Khan University Hospital, Karachi, PAK; 3 Surgery, The Aga Khan University, Karachi, PAK

**Keywords:** covid-19, guidelines, management, surgery, pandemic

## Abstract

Background

The coronavirus disease 2019 (COVID-19), declared a pandemic in March 2020, has affected the entire healthcare system, including the surgical practice. Guidelines for the management of surgical patients during this COVID-19 era need to be established to provide timely yet safe surgical care. In this study, we aimed to evaluate the outcomes of the COVID-19 testing algorithm established for surgery patients presenting to a tertiary care hospital in Karachi, Pakistan, and to compare the outcomes among patients who underwent elective versus emergency surgery.

Methodology

This is a cross-sectional study conducted at a tertiary care hospital in Pakistan to apply and assess the outcomes of the COVID-19 testing algorithm established for patients presenting for surgery. We included all patients who underwent any surgery from May to October 2020. The total sample size was 6,846. The data were analyzed using SPSS version 23 (IBM Corp., Armonk, NY, USA). The categorical variables were assessed using the chi-square or Fisher’s exact test. A p-value of <0.05 was considered significant.

Results

A total of 6,846 surgeries were performed from May 1 to October 31, 2020. In total, 74% of the surgeries were elective procedures. We observed that a significantly higher proportion of emergency surgery patients tested positive for COVID-19 (4.2%) compared to elective surgery patients (25/5,063, 0.5%). A higher proportion of surgeries were performed in September (1,437, 21%) and October (1,445, 21%) while the lowest number of surgeries were performed in May (625, 9.1%). From week one to week five, a higher proportion of emergency surgeries were performed (32%) compared to elective surgeries (25%). Only 1.9% of the patients who were undergoing surgery were COVID-19 positive, with the highest number of COVID-19 cases presenting in June. Overall, 74 (4.2%) of the COVID-19-positive patients underwent emergency surgeries.

Conclusions

The timely establishment of well-defined guidelines for surgical management during the pandemic allowed us to provide timely and effective surgical care to patients with the priority of minimizing the spread of COVID-19 and preventing unnecessary deferral of surgeries.

## Introduction

The disease declared as coronavirus disease 2019 (COVID-19) by the World Health Organization (WHO) was initially known as severe acute respiratory syndrome coronavirus 2 (SARS-CoV-2) [1]. It is caused by a single-stranded RNA virus that belongs to the coronavirus family known as 2019-nCoV (SARS-CoV-2). The disease is highly contagious and is transmitted mainly by droplets or close contact [2]. The first case occurred in Wuhan, Hubei Province, China in December 2019 [3]. On March 11, 2020, the WHO declared COVID-19 a pandemic, and by March 26, 2020, it had spread to almost 199 countries and territories worldwide with more than 462,680 cases and around 20,834 deaths [4]. The COVID-19 pandemic reached Pakistan on February 26, 2020, when a student in Karachi tested positive upon returning from Iran. By March 18, cases had been registered in all four provinces [5]. In Punjab, there were 1,493 cases, Sindh 881 cases, Khyber Pakhtunkhwa 405 cases, Balochistan 191 cases, Gilgit Baltistan 210 cases, Azad Jammu and Kashmir 15 cases, and Federal (ICT) 82 cases for a total of 3,277 cases [5].

With the increasing incidence of COVID-19 in different parts of the world, the COVID-19 pandemic has disrupted healthcare services, especially their capability to manage affected people and the ability to provide standard treatment for critically ill patients in a safe environment [2]. Similarly, surgical procedures have been minimized or temporarily suspended to address the overwhelming and devastating increase in COVID-19 patient care needs [6]. However, decisions regarding surgical intervention in this resource-scarce time need rigorous ethical and clinical evaluation [7]. In this pandemic era, all healthcare providers must follow standardized essential perioperative measures including the use of personal protective equipment (PPEs) to control disease transmission and avoid unwanted complications. All patients need to be managed as COVID-19 patients until confirmed by testing. Elective procedures should be postponed, and only urgent, lifesaving procedures and oncologic surgeries should be performed that are associated with worse outcomes if delayed [2].

In a lower-middle-income country (LMIC) such as Pakistan with limited healthcare facilities, the effect of COVID-19 has been severe. Effective management and implementation of processes and planning are imperative [7]. To perform surgical procedures, additional time is required to prepare for surgery in a suspected/confirmed case of COVID-19 in daily routines regardless of whether surgery would happen. Therefore, for effective management of surgical procedures, a tertiary care hospital in Karachi, Pakistan established a COVID-19 testing algorithm for effective management of surgical patients.

Objectives

In this study, we aimed to evaluate the outcomes (i.e., COVID-19-positive cases according to the type of code and proportion of surgeries deferred) of a COVID-19 testing algorithm established for surgery patients presenting to a tertiary care hospital in Karachi, Pakistan. In addition, we compared the outcomes of patients who underwent elective versus emergency surgery.

## Materials and methods

This cross-sectional study was conducted in the Department of Surgery of a tertiary care hospital in Karachi, Pakistan. Non-probability consecutive sampling technique was employed to recruit patients from May to October 2020. All patients presenting for surgical procedures from May to October 2020 were included. Patients who left against medical advice or with missing information were excluded.

Description of the COVID-19 testing algorithm for surgery patients

According to the COVID-19 testing algorithm (Appendix 1) for surgery patients, patients were screened for COVID-19 using the Patient Under Investigation (PUI) tracking form by a nurse. This form recorded all COVID-19-related information of the patient. The form included the patients’ demographics and history of any of the following symptoms within 14 days: fever, cough, shortness of breath, myalgia, and sore throat. Moreover, it also included any history of travel from another country, direct contact with a person with confirmed/probable COVID-19, healthcare worker returning from a congregation, and engagement in public dealing.

Black code refers to an elective surgery or elective procedure that is scheduled in advance because it does not involve a medical emergency. Orange code refers to an urgent surgery that can wait until the patient is medically stable but should generally be done within two days. Red code refers to an emergency surgery that must be performed without delay; in this code, the patient has no choice other than immediate surgery if permanent disability or death is to be avoided.

According to this algorithm, for black code elective cases, in a non-suspected COVID-19 patient, the testing is done at the COVID-19 test center. Patients with low to medium risk or those who are asymptomatic are sent for pooled COVID-19 polymerase chain reaction (PCR) test. However, patients with high risk are sent for individual COVID-19 PCR testing. Patients with a negative COVID-19 result proceed with the surgery. For patients with a positive COVID-19 result, surgery is either deferred, or if the surgery cannot be deferred, they undergo surgery in a negative pressure flow operating room (OR) with full PPE (N95 masks and face and eye shields). Following the surgery, they are admitted to the COVID-19 Diagnostic and Treatment Zone (CDTZ). However, if the patient is a COVID-19-suspected case at baseline, surgery is either deferred, or if it cannot be deferred, they are admitted to the CDTZ.

For orange code emergency cases, the same procedure of COVID-19 screening is followed and the tracking PUI form is documented by the nurse. COVID-19-suspected cases are admitted in the CDTZ (under the care of the primary team) and non-suspected cases are admitted in private rooms. COVID-19 testing is done in a designated inpatient area by trained surgery residents or nurses with appropriate PPE, including N95 masks and face and eye shields. Those who test negative are admitted and proceed with surgery as indicated. For those who test positive, the surgery is either deferred or patients undergo surgery in a negative pressure flow OR with full PPE (N95 masks and face and eye shields) and are admitted to the CDTZ after surgery.

For red code emergency cases, the COVID-19 test is performed in the emergency room (ER) or the OR. These patients undergo surgery in a negative pressure flow OR with full PPE (N95 masks and face and eye shields) and are admitted to the CDTZ if the COVID-19 test result is not available by the time surgery concludes. Those who test negative are shifted to the service line bed while those who test positive remain in the CDTZ after surgery.

The data for the study were collected on a Proforma from the patients’ medical records during May and October 2020. Data regarding patients’ demographic, type of case (elective, orange, or red), type of surgery, COVID-19 screening (non-suspected or suspected case), COVID-19 results (positive or negative), surgery outcome, and classification of surgery according to specialties were collected.

Because there was no interaction with the patients and all the information was collected from hospital medical records, an ethical exemption was sought from the Aga Khan University Ethical Review Committee (ERC #2020-5014-11563). This study is registered in clinicaltrial.gov NCT04911868. All patient information was kept securely in lock and key. The database was password protected and only accessible to the research team. All the patient information was de-identified. When patients were admitted to the hospital consent was taken from them. They were informed that their information will be used for research purposes and the study results will be published in group form and no individual information will be disclosed.

The data were analyzed using SPSS version 22 (IBM Corp., Armonk, NY, USA). Descriptive for qualitative variables was reported as frequency and percentages and was assessed using the chi-square and Fisher’s exact test. Bar charts for categorical variables and histogram/line graphs were presented for quantitative variables. A p-value of <0.05 was kept significant throughout the study. This work has been reported in line with the Strengthening The Reporting Of Cohort Studies in Surgery (STROCSS) criteria [8].

## Results

**Figure 1:**
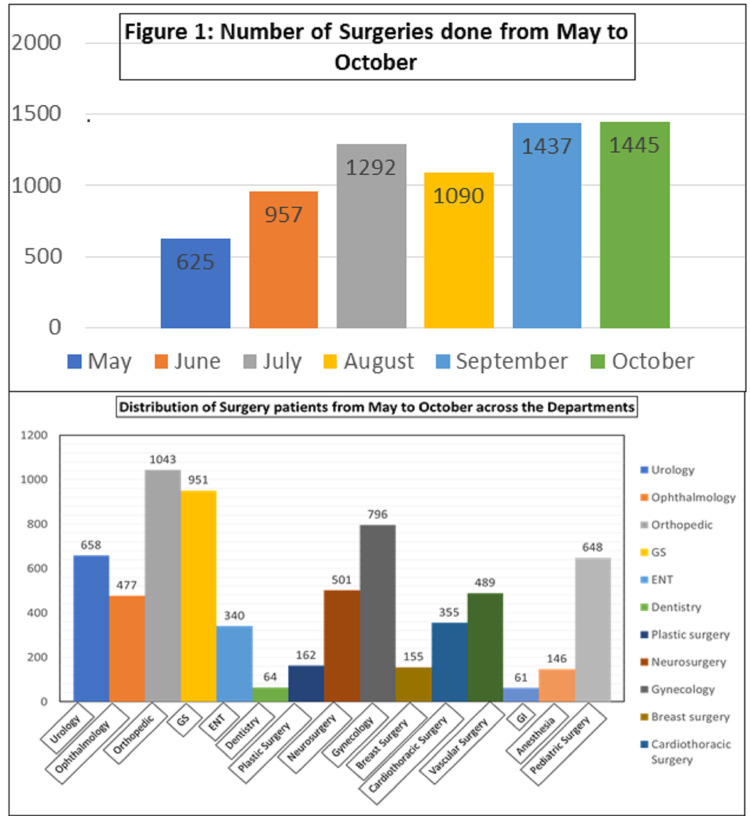
Number of surgeries and distribution of patients from May to October 2020. GS: general surgery; ENT: ear, nose, throat

A total of 6,846 surgeries were performed from May to October 2020. Table 1 shows the COVID-19 status of surgery patients and the types of surgeries patients underwent from May to October 2020. A higher proportion of surgeries were performed in September (1,437, 21%) and October (1,445, 21%), while the lowest number of surgeries were performed in May (625, 9%). The orthopedic section performed the highest number of surgeries (1,043, 15%), followed by general surgery (951, 14%), while the least number of surgeries were performed by the dentistry section (<1%) (Figure 1). A higher proportion of elective surgeries (5,063, 74%) were performed compared to emergency surgeries (1,783, 26%) during this time period. Approximately 99 patients (1.4%) tested positive for COVID-19.

**Table 1:** Description of the COVID-19 status and type of surgeries from May to October 2020. COVID-19: coronavirus disease 2019; GS: general surgery; ENT: ear, nose, throat; GI: gastrointestinal; BLK: nerve block; EPI: epidural anesthesia; G/A: general anesthesia; IV/S: intravenous sedation; L/A: local anesthesia; MAC: monitored anesthesia care; SP: spinal anesthesia

		N (%)
	Total	6,846
Type of surgery	Elective	5,063 (74)
	Emergency	1,783 (26)
Color code	Black	5,071 (74.1)
	Orange	1,479 (21.6)
	Red	296 (4.3)
COVID-19 status	Positive	99 (1.4)
	Negative	5,610 (81.9)
	Non-applicable	1,137 (16.6)
Month of surgeries	May	625 (9.1)
	June	957 (14)
	July	1,292 (18.9)
	August	1,090 (15.9)
	September	1,437 (21)
	October	1,445 (21.1)
Speciality	Urology	658 (9.6)
	Ophthalmology	477 (7)
	Orthopedic	1,043 (15.2)
	GS	951 (13.9)
	ENT	340 (5.0)
	Dentistry	64 (0.9)
	Plastic surgery	162 (2.4)
	Neurosurgery	501 (7.3)
	Gynecology	796 (11.6)
	Breast surgery	155 (2.3)
	Cardiothoracic	355 (5.2)
	Vascular	489 (7.1)
	GI	61 (0.9)
	Anesthesia	146 (2.1)
	Pediatrics	648 (9.5)
Anesthesia type	BLK	52 (0.8)
	CS	1 (0.02)
	EPI	2 (0.03)
	G/A	5,250 (76.7)
	IV/S	1 (0.03)
	L/A	1,122 (16.4)
	MAC	59 (0.9)
	SP	336 (4.9)
	Topical	23 (0.3)

**Figure 2:**
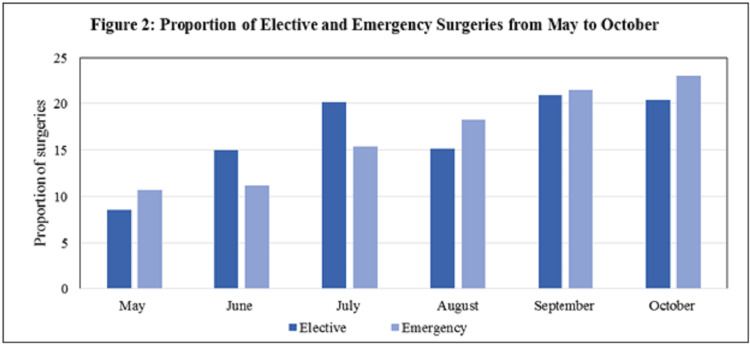
Proportion of elective and emergency surgeries from May to October 2020.

Figure 2 indicates the proportion of elective and emergency surgeries performed from May to October 2020. We observed that in May emergency surgeries were significantly higher (11%) compared to elective surgeries (9%). The proportion of elective surgeries increased in June (15%) and July (20%) compared to emergency surgeries. However, from August to October, there was an increase in emergency surgeries compared to elective surgeries.

**Figure 3:**
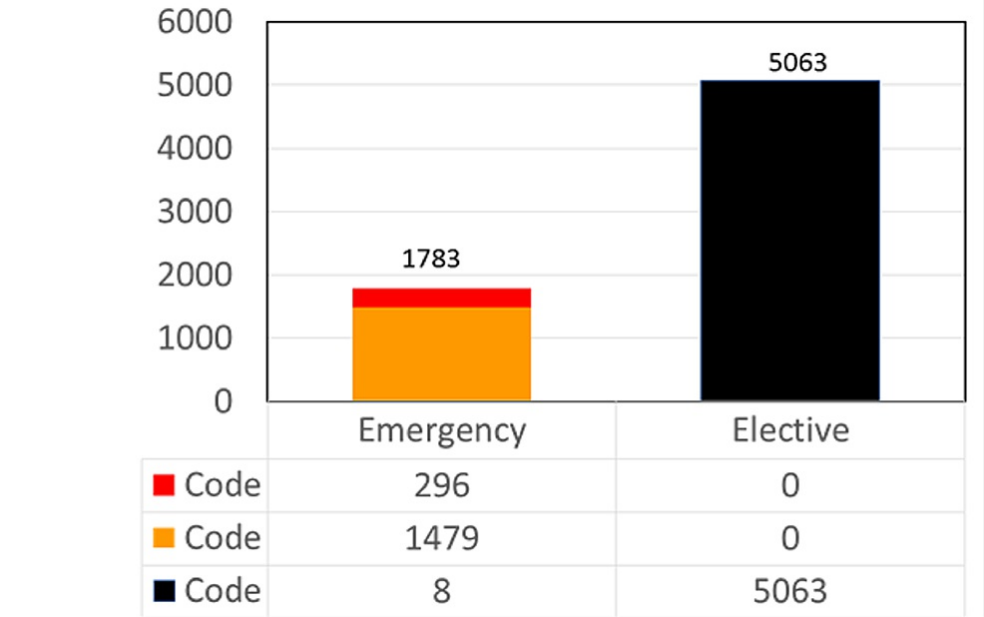
Distribution of elective and emergency surgeries.

Table 2 indicates the COVID-19 status of surgery patients and the type of surgery. We observed that 100% of the black code patients underwent elective surgery. However, the majority of the patients who underwent emergency surgery were of the orange code (83%), 17% of the red code, and <1% of the black code (Figure 3). We observed that a significantly higher proportion of emergency surgery bookings were from the general surgery section (18%). However, a significantly higher proportion of elective surgery bookings were from the urology section (12%) and the ophthalmology section (8%). A significantly higher proportion of patients presenting for emergency surgeries were positive for COVID-19 (4%) compared to those presenting for elective surgeries (<1%).

**Table 2:** Patients’ COVID-19 status and the type of surgery. COVID-19: coronavirus disease 2019; GS: general surgery; ENT: ear, nose, throat; GI: gastrointestinal

		Type of surgery		
		Elective (n = 5,063)	Emergency (n = 1,783)	P-value
		n(%)	n(%)	
Colour code	Black (5,071)	5,063 (100)	8 (0.4)	<0.001*
	Orange (1,479)	0	1,479 (83)	
	Red (296)	0	296 (16.6)	
COVID-19 status	Positive (99)	25 (0.5)	74 (4.2)	<0.001*
	Negative (5610)	411 (81.2)	1,498 (84.0)	
	Not applicable (1,137)	926 (18.3)	211 (11.8)	
Specialty	Urology (658)	623 (12.3)	35 (2.0)	<0.001*
	Ophthalmology (477)	417 (8.2)	60 (3.4)	
	Orthopedic (1,043)	735 (14.5)	308 (17.3)	
	GS (951)	628 (12.4)	323 (18.1)	
	ENT (340)	299 (5.9)	41 (2.3)	
	Dentistry (64)	58 (1.1)	6 (0.3)	
	Plastic surgery (162)	98 (1.9)	64 (3.6)	
	Neurosurgery (501)	336 (6.6)	165 (9.3)	
	Gynecology (796)	548 (10.8)	248 (13.9)	
	Breast surgery (155)	145 (2.9)	10 (0.6)	
	Cardiothoracic (355)	281 (5.6)	74 (4.2)	
	Vascular (489)	322 (6.4)	167 (9.4)	
	GI (61)	26 (0.5)	35 (2)	
	Anesthesia (146)	140 (2.8)	6 (0.3)	
	Pediatrics (648)	407 (8)	241 (13.5)	

**Figure 4:**
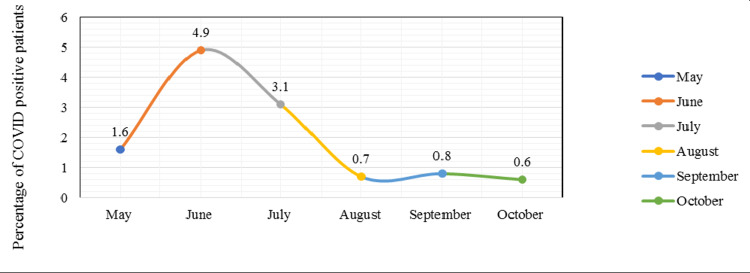
COVID-19 positivity status of surgery patients from May to October 2020. COVID-19: coronavirus disease 2019

Figure 4 shows the COVID-19 positivity status of surgery patients from May to October 2020. In May, of the 625 surgery patients, <1% tested positive for COVID-19. In June, a significant rise in COVID-19-positive cases was observed; of the 957 surgery patients, 4% tested positive for COVID-19. However, from August to October, there was a decline in COVID-19 positivity to <1% among surgery patients.

Table 3 indicates the COVID-19 status of the surgery patients. We observed that a significantly higher proportion of emergency surgery patients tested positive for COVID-19 (74/1,783, 4%) compared to elective surgery patients (25/5,063, <1%). Moreover, a significantly higher proportion of surgery patients of red code tested positive for COVID-19 (49/296, 18%) compared to the orange code (2%) and black code (<1%). In addition, a significantly higher proportion of patients who underwent gynecological surgery tested positive (57/796, 7%) compared to other specialties.

**Table 3:** COVID-19 status of surgery patients. *Significant at p-value < 0.05 using the chi-square/Fisher’s exact test. **Highest proportion of COVID-19-positive patients. COVID-19: coronavirus disease 2019; GS: general surgery; ENT: ear, nose, throat; GI: gastrointestinal; EPI: epidural anesthesia; G/A: general anesthesia; L/A: local anesthesia; MAC: monitored anesthesia care; SP: spinal anesthesia

		COVID-19 positive (n = 99)	P-value
Type of surgery	Elective (5,063)	25 (0.5)	<0.001*
	Emergency (1,783)	74 (4.2)**	
Color code	Black (5,071)	25 (0.6)	<0.001*
	Orange (1,479)	25 (1.9)	
	Red (296)	49 (17.7)**	
Specialty	Urology (658)	1 (0.2)	<0.001*
	Ophthalmology (477)	4 (0.8)	
	Orthopedic (1,043)	6 (0.6)	
	GS (951)	8 (0.8)	
	ENT (340)	3 (0.9)	
	Neurosurgery (501)	3 (0.6)	
	Gynecology (796)	57 (7.2)**	
	Breast surgery (155)	1 (0.6)	
	Cardiothoracic (355)	1 (0.3)	
	Vascular (489)	9 (1.8)	
	GI (61)	3 (4.9)	
	Pediatrics (648)	3 (0.5)	
Anesthesia type	EPI (2)	1 (50)	<0.001*
	G/A (5,249)	55 (1)	
	L/A (1,122)	5 (0.4)	
	MAC (59)	1 (1.7)	
	SP (336)	37 (11)	

## Discussion

The COVID-19 pandemic has placed unprecedented demands on the healthcare system. Although surgical services have been affected indirectly, they have not been spared. Organizations such as the Centers for Disease Control and Prevention (CDC), WHO, American College of Surgeons have recommended postponing/rescheduling elective surgeries to prevent the spread of the virus [9-11]. The cancellation or deferral of elective surgeries has created a backlog of surgery patients. An estimated 28,000 procedures were canceled or postponed during the peak 12 weeks of the pandemic [12]. There is no consensus on how non-emergency procedures should proceed and under what circumstances. The uncertainty of the duration of the COVID-19 pandemic implies that patients may be deprived of timely surgical care. Deferral of surgical procedures is a temporary measure, and a more long-term plan is needed to reintroduce elective surgery with the challenge of minimizing the risk of patients and the surgical staff contracting the infection while providing patients with the required surgical care [13]. Therefore, a proper framework is needed to prioritize surgical patients keeping in mind their COVID-19 status, the urgency of the procedure, risk of possible infection, and the risk of deferring the procedure. The COVID-19 testing algorithm was established by a tertiary care hospital to ensure the safe and effective management of surgical patients during the COVID-19 pandemic.

The first COVID-19 case in Pakistan was diagnosed in March 2020, and the number of cases peaked in June and July 2020 with a downward trend thereafter [14]. This was consistent with the number of COVID-19-positive cases presenting for surgery at our center, with the highest number presenting in June 2020, followed by a decrease in the number of COVID-19-positive patients in the following months.

This study found that the number of elective surgeries performed was the lowest in May 2020, with the majority of surgeries performed during this time being emergency procedures. Elective surgeries were postponed/deferred due to an increase in COVID-19 cases in May according to our surgical management guidelines. Our management recommendations were consistent with those stated in the literature, in that elective operations should be deferred, and only urgent, life-saving procedures and oncologic surgeries should be performed that are associated with worse outcomes if delayed [2]. The number of elective surgeries performed increased in June and July 2020, despite the increasing number of COVID-19 cases. This can be attributed to the institution’s adaptation to the challenges and the “new normal” of the pandemic. Further, by the time COVID-19 cases peaked, effective guidelines had been established to help ensure the safety of both the surgical patients and the surgical teams. Surgical intervention decisions must be subjected to strict ethical and clinical scrutiny during this period with limited resources [7].

All the cases presenting for elective surgery were labeled as black code while the majority of cases presenting for emergency surgery were labeled as orange code followed by red code. Following the aforementioned algorithm, only 1.4% of the patients who underwent surgery were positive for COVID-19. The majority of these patients underwent emergency surgery, and a greater proportion belonged to the red code. Surgeries on COVID-19 patients were performed in negative pressure flow OR with full PPE (including N95 masks and face and eye shields). Such patients were then admitted to the CTDZ. Our surgical management algorithm followed the guidelines similar to those from high-volume facilities in high-income countries (HICs) that include patient triaging based on symptoms and disease; patient segregation based on COVID-19 status; PPE use based on the WHO recommendations; limiting aerosolizing procedures in the OR; use of negative-pressure ORs for COVID-19-positive patients; use of negative-pressure intensive care units for postoperative patients (in case these patients develop COVID-19 infection); and immediate postoperative step-down areas [15]. The practice patterns reported in LMICs are similar to those recommended by HICs; however, three facilities in the Philippines reported having no negative-pressure OR, demonstrating the economic discrepancy [16-18]. In the absence of such facilities, in some studies, numerous directed air-conditioning systems were used to divert airflow away from the OR [19]. When operating on COVID-19-positive patients in LMICs, the emphasis needs to be placed on PPE use rather than the OR setting, unlike in HICs [17,18,20,21].

To our knowledge, this is the first study from Pakistan describing a COVID-19 testing algorithm in surgery patients. There are some limitations of the study. Postsurgical testing was not done and the spread of COVID-19 during hospitalization for surgery was not assessed. Moreover, the survival rates and length of hospital stay of the patients who had COVID-19 while undergoing surgery were not available and therefore not reported in this study.

## Conclusions

Our management recommendations are in line with those reported in the literature, suggesting that urgent, life-saving procedures and oncologic surgeries with worse outcomes should be considered timely. During the pandemic, we were able to provide timely and effective surgical care to patients by establishing well-defined surgical management guidelines that were consistent with the guidelines recommended by HICs. This allowed us to prioritize preventing the spread of COVID while also avoiding unnecessary surgery deferral. Other LMICs can use a similar algorithm to establish a surgical management strategy during the pandemic.

## Figures and Tables

**Figure 5:**
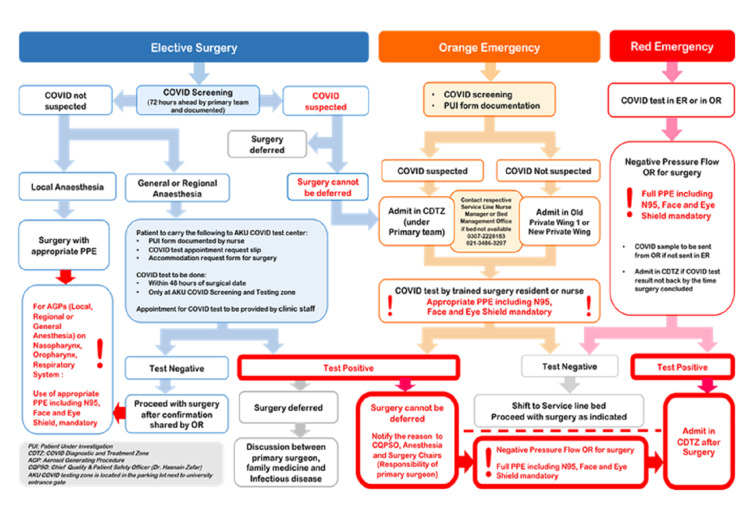
COVID-19 testing algorithm for surgery patients. COVID-19: coronavirus disease 2019; PPE: personal protective equipment; OR: operating room; ER: emergency room; CTDZ: COVID-19 treatment and diagnostic zone
